# Prognostic nomogram for predicting long-term cancer-specific survival in patients with lung carcinoid tumors

**DOI:** 10.1186/s12885-021-07832-6

**Published:** 2021-02-08

**Authors:** Yanqi He, Feng Zhao, Qingbing Han, Yiwu Zhou, Shuang Zhao

**Affiliations:** 1 Department of Respiratory and Critical Care Medicine, West China Hospital, Sichuan University, Chengdu, China; 2 Department of Cancer Center, Sichuan Academy of Medical Sciences&Sichuan Provincial People’s Hospital, Chengdu, China; 3 Department of Emergency Medicine, Emergency Medical Laboratory, West China Hospital, Sichuan University, Chengdu, Sichuan China; 4 Disaster Medical Center, Sichuan University, Chengdu, Sichuan China

**Keywords:** Lung carcinoid tumors, Nomogram, Cancer-specific survival

## Abstract

**Background:**

Lung carcinoid is a rare malignant tumor with poor survival. The current study established a nomogram model for predicting cancer-specific survival (CSS) in patients with lung carcinoid tumors.

**Methods:**

A total of 1956 patients diagnosed with primary lung carcinoid tumors were extracted from the Surveillance, Epidemiology, and End Results database. The specific predictors of CSS for lung carcinoid tumors were identified and integrated to build a nomogram. Validation of the nomogram was conducted using parameters concordance index (C-index), calibration plots, decision curve analyses (DCAs), and the receiver operating characteristic (ROC) curve.

**Results:**

Age at diagnosis, grade, histological type, N stage, M stage, surgery of the primary site, radiation of the primary site, and tumor size were independent prognostic factors of CSS. High discriminative accuracy of the nomogram model was shown in the training cohort (C-index = 0.873), which was also testified in the internal validation cohort (C-index = 0.861). In both cohorts, the calibration plots showed good concordance between the predicted and observed CSS at 3, 5, and 10 years. The DCA showed great potential for clinical application. The ROC curve showed superior survival predictive ability of the nomogram model (area under the curve = 0.868).

**Conclusions:**

We developed a practical nomogram that provided independent predictions of CSS for patients with lung carcinoid tumors. This nomogram may have the potential to assist clinicians in prognostic evaluations or developing individualized therapies for patients with this neoplasm.

## Background

Lung carcinoid is a rare malignant tumor, accounting for 1–2% of all primary lung malignancies, with an estimated annual incidence of 2.3–2.8 cases per million [1, 2]. The incidence of lung carcinoid tumor has increased sharply in the past three decades, partly due to the broad application of cross-sectional imaging and bronchoscopy in population screening [3]. Lung carcinoid is histologically classified as typical carcinoids (TCs) and atypical carcinoids (ACs), characterized by a low mitotic count with the absence of necrosis. TCs show fewer than two mitoses per 2 mm^2^ of viable tumor and lack necrosis, while ACs have 2–10 mitoses per 2 mm^2^ and/or foci of necrosis [4]. TCs and ACs exhibit significant variation in their malignant behaviors. TCs usually have a good prognosis, presenting with a low rate of nodal involvement or distant metastasis, whereas in contrast, ACs are more aggressive with a higher rate of occurrence in both local or distant tumor spread and worse prognosis [5, 6].

Heterogeneous biological behaviors of lung carcinoid tumors has made the prediction of prognosis challenging. Cardillo et al. [7] reported that the prognosis of patients with lung carcinoid tumors is more influenced by nodal status than histological subtype. In N0 and N1 patients, no statistically significant difference has been found between TCs and ACs. However, N2 bronchial carcinoid tumors show a dismal prognosis. Maurizi et al. [8] showed that histological subtypes can only impact disease-free survival with a statistically significant advantage for TCs. ACs have a higher propensity to develop local recurrence and distant recurrence [9, 10]. Pathologic lymph node metastasis is another important predictor of survival in patients with lung carcinoid tumors. Lymph node metastasis is associated with decreased survival for both TC and AC subtypes with a tumor size greater than 2 cm. However, lymph node involvement is not associated with survival in patients with small TCs (tumor size < 2 cm) [11]. Detection of patients under high risk with homogenous prognosis especially at an early stage could be helpful to improve clinical practice and decision-making.

The tumor-node-metastasis (TNM) classification system has been utilized for tumor staging in lung carcinoids since 2010, which is also now recommended by the North American Neuroendocrine Tumor Society and European Neuroendocrine Tumor Society [12, 13]. However, TNM staging is not a perfect predictor of lung carcinoid tumor prognosis, because it does not take into account the effect of tumor size, metastasis sites/patterns, pathological, genetic, and therapeutic factors, which are significantly associated with prognosis. Peter et al. [11] demonstrated that lymph node metastases were associated with poor survival in larger TCs greater than 2 cm in size, but had no significance in patients with smaller TCs less than 2 cm [11]. It has been shown that AC patients treated with surgery have reduced risk of death, whereas radiation treatment is associated with an increased risk of death [14]. Wedge resection is likely to be equal to segmental resection for TC patients at the localized stage [15]. It has also been reported that Black race and older age are associated with a higher rate of disease-specific mortality [16]. Although previous studies have suggested several potential predictors of lung carcinoid survival, none have been confirmed in a large-scale dataset, and no prognostic model has been established based on a valid predictor.

Nomograms have been widely used as prognostic tools to predict disease outcomes. The advantage of this model includes a simple and visualization figure that cover many relevant variables, which have been improved the discriminatory accuracy of outcome predictions [17, 18], and have been widely used to quantify the risk of various malignancies [19, 20]. To the best of our knowledge, no previous study has built nomogram model for patients with lung carcinoid tumors. Therefore, in this study we developed an elaborate nomogram to assess prognosis in patients with lung carcinoid tumors in terms of 3-, 5-, and 10-year cancer-specific survival (CSS) using data from the Surveillance, Epidemiology, and End Results (SEER) database [21].

## Methods

### Study patients and study design

Data were retrieved from 18 population-based cancer registries in the SEER program using SEER*Stat software (version 8.3.6) [22]. The SEER program is sponsored by the National Cancer Institute, covering approximately 30% of the United States population. Among all patients with lung cancer, we selected TC (ICD-O-3 code: 8240) and AC (ICD-O-3 code: 8249) bronchopulmonary carcinoid tumors (primary site C340–C343, C348, and C349) diagnosed between 1975 and 2016. Patients with only one primary cancer in their lifetime were chosen. Patient informed consent was waived as patients were de-identified and all data are publicly available. Demographic data (including age at diagnosis, gender, race, marital status, survival status and times), tumor characteristics (primary site, laterality, histologic grade, histologic type, primary tumor size, T, N, and M stages), and treatment information (surgery, radiation and chemotherapy) were all obtained from the SEER database. The exclusion criteria were missing or incomplete data and diagnosis of carcinoid tumors at the time of autopsy or on the death certificate. CSS was defined as death from lung carcinoid tumors according to the SEER database. The main outcome of this study was CSS, which was the interval between the diagnosis of carcinoid tumors and the occurrence of cancer-specific death.

### Statistical analyses

Statistical analyses were performed using R software version 3.5.1 (R Foundation for Statistical Computing, Vienna, Austria) and SPSS version 20.0 (IBM Corporation, Armonk, NY, USA). Cox proportional hazards regression models were performed to identify independent prognostic factors of survival, by calculating hazard ratios and corresponding 95% confidence intervals. Variables that were significant (*p* < 0.05) in univariate Cox regression analyses were included in the multivariate Cox regression analysis. Variables that obtained statistically significance in multivariate models were finally included in the nomogram analysis. We assigned each finally included variable a score ranging from 0 to 100 based individual patient’s demographic and clinical characteristics. Total score was calculated by summing all individual score. Survival rates of 3, 5, and 10 years were also obtained from the nomogram. Adequate discrimination and calibration were performed to test and validate the prognostic accuracy of the nomogram model [23]. Discrimination was quantified using Harrell’s concordance index (C-index), in which an absolute value close to 1 indicates that a nomogram model has strong predictive ability. The nomogram was further subjected to bootstrapping validation (1000 bootstrap replicates) to calculate the relatively corrected C-index. Calibration plots were developed to evaluate predictive accuracy and to assess the concordance between predicted and observed ongoing survival probabilities. Clinical usefulness of the novel nomogram was assessed through decision curve analyses (DCAs), and meanwhile Kaplan-Meier curves and the log-rank test were applied to illustrate and compare the CSS of patients from different risk groups. The precision of the survival predictions was also evaluated using the area under the receiver operating characteristic (ROC) curve. A two-sided *P* < 0.05 indicated statistical significance.

## Results

### Characteristics of patients

A total of 1956 patients who met the inclusion criteria and had complete information were identified from the SEER database. Table 1 shows the baseline demographic and clinicopathological characteristics. The median age of all patients was 60 years (range, 21–95). More than half of the patients were diagnosed at 60 years of age and older. The majority of the patients were White (89.2%), women (66.6%) and TC (87.0%). The most common primary sites were lower lobe (40.5%) and upper lobe (31.7%). More than half of the laterality was right (58.4%). The most frequent primary tumor size was between 20 and 40 mm (44.6%), followed by tumor size < 20 mm (43.0%). In all, 74.2% of patients had well-differentiated tumors and 71.0% of patients were categorized with localized disease. Among atypical patients, 25.6% of patients had well-differentiated tumors and 46.1% of patients were categorized with localized disease. Regarding treatment, 88.5% of patients were managed by surgery, only 5.6% received radiation treatment, and only 5.8% received chemotherapy treatment. Of the patients who were treated with surgery, 63.1% had a lobectomy resection, whereas 13.5% had wedge resection.

**Table 1:** Demographics and clinicopathological characteristics of lung carcinoid tumors

	All (*N* = 1956)
**Age at diagnosis, years**	
< 60	952 (48.7%)
≥ 60	1004 ((51.3%)
**Gender**	
Female	1302 (66.6%)
Male	654 (33.4%)
**Race**	
White	1745 (89.2%)
Other	211 (10.8%)
**Insurance**	
Yes	1587 (81.1%)
No	369 (18.9%)
**Marital status**	
Married	1140 (58.3%)
Unmarried	816 (41.7%)
**Primary site**	
Main bronchus	108 (5.5%)
Upper lobe, lung	621 (31.7%)
Middle lobe, lung	337 (17.2%)
Lower lobe, lung	792 (40.5%)
Overlapping lesion of lung	37 (1.9%)
Lung, NOS	61 (3.2%)
**Grade**	
Well	1451 (74.2%)
Moderately/Poorly/Undifferentiated	505 (25.8%)
**Laterality**	
Right	1142 (58.4%)
Other	814 (46.4%)
**Historic Stage**	
Localized	1389 (71.0%)
Regional/Distant/Unstaged	567 (29.0%)
**Histological type**	
Typical carcinoid	1702 (87.0%)
Atypical carcinoid	254 (13.0%)
**T stage**	
T1	1133 (57.9%)
T2-T4	823 (42.1%)
**N stage**	
N0	1622 (83.0%)
N1-N3	334 (17.0%)
**M stage**	
M0	1814 (92.7%)
M1	142 (7.3%)
**Surgery**	
No	225 (11.5%)
Lobectomy	1235 (63.1%)
Local	119 (6.1%)
Wedge	264 (13.5%)
Pneumonectomy	103 (5.3%)
NOS	10 (0.5%)
**Radiation**	
No	1847 (94.4%)
Yes	109 (5.6%)
**Chemotherapy**	
No	1843 (94.2%)
Yes	113 (5.8%)
**Radiation after surgery**	
No	1898 (97.0%)
Yes	58 (3.0%)
**Tumor size**	
< 20 mm	841 (43.0%)
20–40 mm	873 (44.6%)
> 40 mm	242 (12.4%)

### Prognostic factors of CSS

The univariate and multivariate results of prognostic factors for CSS of patients with lung carcinoid tumors are shown in the Table 2. In univariable analyses, statistically significant predictive factors of CSS included age at diagnosis (*P* < 0.001), insurance (*P* = 0.002), grade (*P* < 0.001), historic stage (*P* < 0.001), histological type (*P* < 0.001), T Stage (*P* < 0.001), N stage (*P* < 0.001), M stage (*P* < 0.001), surgery of primary site (*P* < 0.001), radiation of primary site (*P* < 0.001), chemotherapy treatment (*P* < 0.001), radiation after surgery (*P* < 0.001), and tumor size (*P* < 0.001). Multivariate analyses only included these prognostic factors with statistical significance in the univariate models. Younger age at diagnosis (*P* < 0.001), having insurance (*P* = 0.001), well-differentiated tumor (*P* < 0.001), TC (*P* = 0.001), N0 stage (*P* = 0.039), M0 stage (*P* = 0.013), and no radiation therapy (*P* = 0.040) were significantly associated with improved CSS among patients with lung carcinoid tumors. Received lobectomy resection (*P* < 0.001), local resection (*P* = 0.010), or pneumonectomy resection (*P* = 0.001) were also significantly associated with improved CSS. Tumor size between 20 and 40 mm (*P* = 0.023), or larger than 40 mm (*P* < 0.001) were negatively associated with CSS among patients with lung carcinoid tumors.

**Table 2:** Univariate and multivariate analysis of each factor’s ability to predict CSS of lung carcinoid tumors

Characteristic	CSS				
	Univariable analysis		Multivariable analysis		
	HR (95% CI)	*p* value	HR (95% CI)		*p* value
**Age at diagnosis**					
< 60	1 (reference)		1 (reference)		
≥ 60	**2.384 (1.714–3.316)**	**0.000**	**2.277 (1.587–3.267)**		**0.000**
**Race**					
White	1 (reference)				
Others	1.389 (0.885–2.179)	0.153			
**Gender**					
Female	1 (reference)				
Male	1.226 (0.894–1.681)	0.205			
**Insurance**					
Yes	1 (reference)		1 (reference)		
No	**1.736 (1.221–2.466)**	**0.002**	**1.850 (1.288–2.658)**		**0.001**
**Marital status**					
Married	1 (reference)				
Un-married	1.072 (0.785–1.465)	0.662			
**Primary site**					
Main bronchus	1 (reference)				
Upper lobe, lung	2.200 (0.954–5.071)	0.064			
Middle lobe, lung	0.680 (0.251–1.839)	0.447			
Lower lobe, lung	1.779 (0.771–4.105)	0.177			
Overlapping lesion of lung	1.511 (0.377–6.050)	0.560			
Lung, NOS	2.754 (0.925–8.205)	0.069			
**Grade**					
Well	1 (reference)		1 (reference)		
Moderately/Pooly/Undifferentiated	**4.48 (3.252–6.171)**	**0.000**	**2.182 (1.537–3.098)**		**0.000**
**Laterality**					
Right	1 (reference)				
Other	1.179 (0.866–1.606)	0.295			
**Historic Stage**					
Localized	1 (reference)		1 (reference)		
Regional/Distant/Unstaged	**4.833 (3.505–6.665)**	**0.000**	1.529 (0.877–2.665)		0.134
**Histological type**					
Typical carcinoid	1 (reference)		1 (reference)		
Atypical carcinoid	**4.767 (3.462–6.564)**	**0.000**	**1.962 (1.340–2.872)**		**0.001**
**T stage**					
T1	1 (reference)		1 (reference)		
T2-T4	**3.228 (2.308–4.514)**	**0.000**	1.142 (0.732–1.784)		0.558
**N stage**					
No	1 (reference)		1 (reference)		
N1-N3	**4.979 (3.658–6.778)**	**0.000**	**1.606 (1.025–2.515)**		**0.039**
**M stage**					
M0	1 (reference)		1 (reference)		
M1	**9.292 (6.648–12.99)**	**0.000**	**1.892 (1.142–3.135)**		**0.013**
**Surgery**					
No	1 (reference)		1 (reference)		
Lobectomy	**0.116 (0.081–0.166)**	**0.000**	**0.332 (0.208–0.532)**		**0.000**
Local resection/destrubition	**0.038 (0.009–0.155)**	**0.000**	**0.148 (0.035–0.627)**		**0.010**
Wedge resection	**0.178 (0.105–0.302)**	**0.000**	0.675 (0.366–1.244)		0.207
Pneumonectomy	**0.020 (0.108–0.382)**	**0.000**	**0.260 (0.122–0.557)**		**0.001**
Surgery, NOS	0.000 (0.000-Inf)	0.993	0.000 (0.000-Inf)		0.993
**Radiation**					
No	1 (reference)		1 (reference)		
Yes	**7.591 (5.331–10.81)**	**0.000**	**1.796 (1.028–3.139)**		**0.040**
**Chemotherapy**					
No	1 (reference)		1 (reference)		
Yes	**7.854 (5.563–11.09)**	**0.000**	1.332 (0.859–2.064)		0.201
**Radiation after surgery**					
No	1 (reference)		1 (reference)		
Yes	**3.981 (2.433–6.515)**	**0.000**	0.667 (0.318–1.401)		0.285
**Tumor size**					
< 20 mm	1 (reference)		1 (reference)		
20-40 mm	**2.102 (1.388–3.184)**	**0.000**	**1.660 (1.071–2.574)**		**0.023**
> 40 mm	**6.473 (4.190–9.999)**	**0.000**	**3.206 (1.867–5.508)**		**0.000**

### Prognostic factors with CSS in subgroup of histological type

We also evaluated prognostic factors in a separate histological type of lung carcinoid tumors (Table 3). Among patients with TC, younger age at diagnosis (*P* < 0.001), female (*P* = 0.039), having insurance (*P* = 0.049), well-differentiated tumor (*P* < 0.001), resection surgery of the primary site (lobectomy resection/wedge resection/pneumonectomy, all *P* < 0.05), no chemotherapy (*P* = 0.020), and tumor size < 20 mm (*P* < 0.000) were independent prognostic factors, and positively associated with improved CSS. In contrast to TC, only younger age at diagnosis (*P* = 0.031), M0 (*P* < 0.000), lobectomy resection (*P* = 0.002), and tumor size < 20 mm (*P* = 0.031) were associated with CSS among patients with AC.

**Table 3:** Univariate and multivariate analysis of each factor’s ability to predict CSS of lung typical carcinoid and atypical carcinoid tumors

Characteristic	Typical Carcinoid				Atypical Carcinoid				
	Univariable analysis		Multivariable analysis		Univariable analysis		Multivariable analysis		
	HR (95% CI)	*p* value	HR (95% CI)	*p* value	HR (95% CI)	*p* value	HR (95% CI)		*p* value
**Age at diagnosis**									
< 60	1 (reference)		1 (reference)		1 (reference)		1 (reference)		
≥ 60	**2.269 (1.497–3.438)**	**0.000**	**2.684(1.710–4.213)**	**0.000**	**1.922 (1.113–3.321)**	**0.019**	**1.975 (1.065–3.660)**		**0.031**
**Race**									
White	1 (reference)				1 (reference)				
Others	1.519(0.862–2.678)	0.148			1.071 (0.509–2.252)	0.857			
**Gender**									
Female	1 (reference)		1 (reference)		1 (reference)				
Male	**1.507(1.013–2.242)**	**0.043**	1.563(1.022–2.390)	**0.039**	0.857 (0.504–1.457)	0.568			
**Insurance**									
Yes	1 (reference)		1 (reference)		1 (reference)				
No	**2.249 (1.431–3.534)**	**0.0004**	**1.622 (1.002–2.626)**	**0.049**	1.346 (0.782–2.317)	0.284			
**Marital status**									
Married	1 (reference)				1 (reference)				
Un-married	0.986 (0.659–1.473)	0.943			1.404 (0.849–2.321)	0.186			
**Primary site**									
Main bronchus	1 (reference)				1 (reference)				
Upper lobe, lung	1.346 (0.569–3.185)	0.498			0.000 (0.000-Inf)	0.996			
Middle lobe, lung	0.681 (0.247–1.878)	0.458			0.000 (0.000-Inf)	0.997			
Lower lobe, lung	1.150 (0.486–2.722)	0.751			0.000 (0.000-Inf)	0.996			
Overlapping lesion of lung	0.508(0.061–4.230)	0.531			0.000 (0.000-Inf)	0.996			
Lung, NOS	1.738(0.489–6.175)	0.393			0.000 (0.000-Inf)	0.996			
**Grade**									
Well	1 (reference)		1 (reference)		1 (reference)		1 (reference)		
Moderately/Pooly/Undifferentiated	**3.537 (2.372–5.272)**	**0.000**	**2.127(1.392–3.250)**	**0.000**	**1.982 (1.006–3.905)**	**0.048**	**1.937(0.947–3.961)**	0.070	
**Laterality**									
Right	1 (reference)				1 (reference)				
Other	1.022(0.686–1.521)	0.915			1.384 (0.844–2.27)	0.198			
**Historic Stage**									
Localized	1 (reference)		1 (reference)		1 (reference)		1 (reference)		
Regional/Distant/Unstaged	**4.016(2.699–5.977)**	**0.000**	1.388 (0.697–2.764)	0.351	**3.749 (2.094–6.712)**	**0.000**	1.882 (0.724–4.893)	0.194	
**T stage**									
T1	1 (reference)		1 (reference)		1 (reference)		1 (reference)		
T2-T4	**3.215 (2.102–4.919)**	**0.000**	1.182 (0.680–2.053)	0.553	**2.487(1.431–4.322)**	**0.001**	1.242 (0.581–2.652)		0.576
**N stage**									
N0	1 (reference)		1 (reference)		1 (reference)		1 (reference)		
N1-N3	**4.753 (3.186–7.092)**	**0.000**	1.630 (0.901–2.948)	0.106	**2.564 (1.55–4.241)**	**0.000**	1.502 (0.719–3.137)		0.279
**M stage**									
M0	1 (reference)		1 (reference)		1 (reference)		1 (reference)		
M1	**7.635 (4.825–12.08)**	**0.000**	1.443 (0.679–3.066)	0.341	**7.527 (4.506–12.57)**	**0.000**	**4.302 (2.150–8.607)**		**0.000**
**Surgery**									
No	1 (reference)		1 (reference)		1 (reference)		1 (reference)		
Lobectomy	**0.121 (0.077–0.188)**	**0.000**	**0.265 (0.140–0.502)**	**0.000**	**0.123 (0.067–0.226)**	**0.000**	**0.299(0.139–0.643)**		**0.002**
Local resection/destrubition	**0.059 (0.014–0.247)**	**0.000**	0.236 (0.053–1.050)	0.058	0.000 (0.000-Inf)	0.996	0.000 (0.000-Inf)		0.995
Wedge resection	**0.075 (0.030–0.192)**	**0.000**	**0.249 (0.090–0.690)**	**0.008**	**0.455(0.229–0.905)**	**0.025**	1.674 (0.677–4.139)		0.264
Pneumonectomy	**0.122 (0.047–0.318)**	**0.000**	**0.121(0.037–0.392)**	**0.000**	**0.336(0.143–0.789)**	**0.012**	0.424(0.142–1.263)		0.123
Surgery, NOS	0.000 (0.000-Inf)	0.995	0.000 (0.000-Inf)	0.994	0.000 (0.000-Inf)	0.999	0.000 (0.000-Inf)		0.999
**Radiation**									
No	1 (reference)		1 (reference)		1 (reference)		1 (reference)		
Yes	**10.84 (6.89–17.05)**	**0.000**	1.512 (0.724–3.156)	0.271	**2.19 (1.241–3.866)**	**0.006**	1.149 (0.573–2.303)		0.696
**Chemotherapy**									
No	1 (reference)		1 (reference)		1 (reference)		1 (reference)		
Yes	**9.244 (5.587–15.3)**	**0.000**	**2.014 (1.118–3.631)**	**0.020**	**2.664 (1.604–4.425)**	**0.000**	0.653 (0.332–1.286)		0.218
**Radiation after surgery**									
No	1 (reference)		1 (reference)		1 (reference)				
Yes	**5.533 (2.847–10.76)**	**0.000**	1.422 (0.520–3.887)	0.492	1.169(0.556–2.454)	0.681			
**Tumor size**									
< 20 mm	1 (reference)		1 (reference)		1 (reference)		1 (reference)		
20-40 mm	**3.028(1.726–5.313)**	**0.000**	**2.310(1.276–4.181)**	**0.006**	0.927 (0.490–1.756)	0.816	0.689(0.332–1.432)		0.184
> 40 mm	**8.470(4.643–15.452)**	**0.000**	**4.286 (2.043–8.993)**	**0.000**	**2.447(1.291–4.640)**	**0.006**	**1.796 (0.757–4.259)**		**0.031**

### Nomogram development and validation

**Fig. 1:**
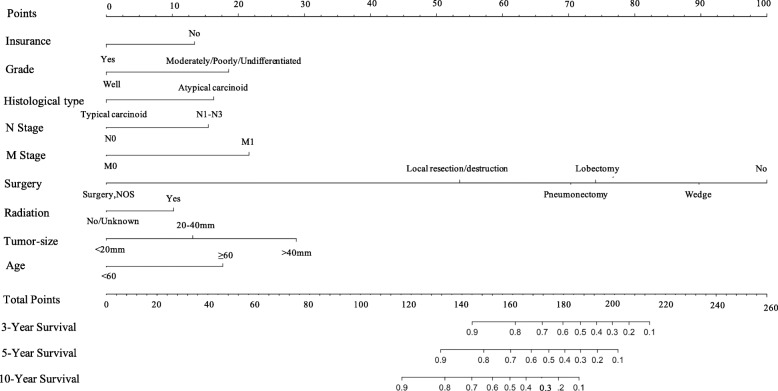
Nomogram predicting 3-, 5-, and 10-year CSS of patients with lung carcinoid tumors. The nomogram summed the points identified on the scale for each variable. The total points projected on the button scale indicate the probabilities of 3-, 5-, and 10-year CSS

**Fig. 2:**
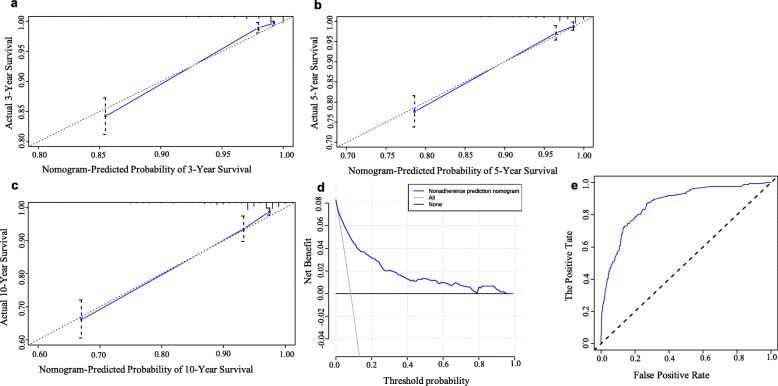
**a–c** Calibration curves of the nomogram for predicting 3-, 5-, and 10-year CSS. Nomogram-predicted CSS is plotted on the x-axis and the actual CSS is plotted on the y-axis. The diagonal dotted line indicates the ideal nomogram, in which the actual and predicted probabilities are identical. The solid line indicates the actual nomogram, of which a closer fit to the dotted line indicates a better calibration. **d**. Decision curves of the nomogram predicting CSS. The x-axis represents the threshold probabilities and y-axis measures the net benefit calculated by adding the true positives and subtracting the false positives. **e**. The ROC of the nomogram for predicting the CSS

Figure 1 shows the nomogram for predicting CSS of lung carcinoid tumors using the significant independent factors that were found in the multivariate analysis. The nomogram showed that the largest contributions to prognosis were resection surgery of primary site and tumor size of primary site, followed by M stage. The C-index for the CSS predictive nomogram was 0.873 and confirmed to be 0.861 through bootstrapping validation. The features of calibration plots for CSS probability at 3, 5, and 10 years indicated that the concordance between predicted and observed survival was optimal (Fig. 2a–c). Furthermore, DCA demonstrated great positive net benefits in the predictive model among nearly all of the threshold probabilities at different time points, which prove the potential clinical values of this model (Fig. 2d). The area under the curve of the nomogram for predicting the CSS rate of lung carcinoid tumors was 0.868, which exhibited superior survival predictive ability of the nomogram model (Fig. 2e).

### Kaplan-Meier analyses

**Fig. 3:**
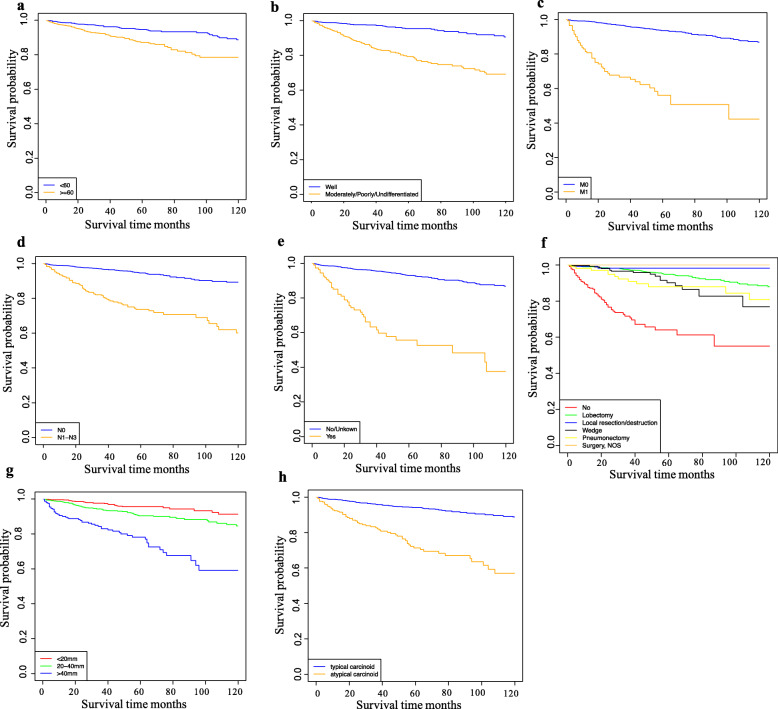
Kaplan-Meier curves of CSS in the training and validation sets by **a** age at diagnosis, **b** grade, **c** M stage, **d** N stage, **e** radiation, **f** surgery of primary site, **g** tumor size, and **h** histological type

With a median (range) follow-up of 126.5 (10–364) months, Kaplan-Meier analysis revealed that the median CSS was not reached. As shown in Fig. 3, worse CSS for patients with lung carcinoid tumors was shown in patients of advanced age (≥60 years), lacking insurance, moderately, poorly, or undifferentiated tumor, AC, N1–N3 stage, M1 stage, radiation therapy, not receiving surgery treatment, and large tumor size (20–40 mm and > 40 mm).

## Discussion

The results of the current study revealed that age at diagnosis, tumor grade, histological type, N stage, M stage, surgery of primary site, radiation of primary site, and tumor size were significant prognostic factors for lung carcinoid tumors. The established nomogram model exhibited high discriminative accuracy and good concordance in the prediction of 3-, 5- and 10-year CSS. Based on results from nomogram, surgery, tumor size, and tumor stage (M stage) were the strongest prognostic predictors for survival rate among patients with lung carcinoid tumors.

The nomogram illustrated that tumor grade was associated with survival among patients with lung carcinoid tumors. Higher CSS has been reported among patients in T1 stage compared to other tumor stages [24, 25]. A worse CSS has been indicated among patients with TCs and stage M1a compared to M0, although it is not statistically significant [26]. Although earlier evidence has recommended that the TNM staging system can be a predictor of prognosis among patient with lung carcinoid tumors, our study failed to detect the predictive value for “T” stage. Our nomogram demonstrated that patients diagnosed with lymph node metastases and distant metastases were associated with higher mortality rate, which was different to results predicting by traditional TNM staging system.

Surgical resection is the only curable therapy for patients diagnosed with lung carcinoid tumors [9]. Surgical resection have been shown to improves disease-free survival and overall survival among patients with lung cancer [27, 28]. We also observed significant differences in CSS treated or not treated with surgical resection, which demonstrated that surgical resection was an independent factor significantly improving in the present cohort. However, the optimal type of surgical treatment for this disease is controversial. To date, many researchers have reached some consensus based on their long-term outcomes of large case series or pool analyses together with cooperating institutions and recommended that lobectomy was the previous surgical techniques of choice, with or without parenchymal resection [6, 29, 30]. ACs could use therapy similar to NSCLCs, i.e. lobectomy or pneumonectomy and systemic lymphadenectomy [31]. Our study also supports the use of lobectomy and pneumonectomy resection improves CSS in in patients with both TCs and ACs. In addition, wedge resection may produce survival outcomes for TC in our results, in accordance with a previous study that supported the use of conservative sublobar resection for TC [32]. Wedge resection is generally considered as a conventional treatment for patients diagnosed with localized TC [15]. Possessing better pulmonary function but a smaller surgical wound will result in better quality of life for operated patients.

Lung carcinoid tumors generally have low proliferative activity and are consequently chemoresistant. To date, no standard chemotherapy regimen is recommended as first-line treatment. Clinical use of triplet or doublet chemotherapy as first-line treatment in lung carcinoid tumors is still controversial. Cumulative evidences have convinced the improved efficacy by using the combination of these chemotherapy regimens. However, the decision is still inconclusive since many of these studies were confronted with small sample size of carcinoid tumors [13, 33, 34]. In the current study, chemotherapy was according to poorer CSS in TC but not in AC. Due to the fact that SEER was not able to provide details of chemotherapy for individual patients, we were not able to conduct subgroup analyses based on chemotherapy regimens.

We also observed overall worse survival among patients who received radiation. This result shall be carefully interpreted. It is noted that radiation is usually provided more critically ill patients who were usually unresectable or had serious comorbidity. Therefore, our findings most likely indicated that patients in a later stage received radiation, rather than radiation therapy was associated with poorer survival.

While tumor size has been previously used as a potential prognosis factor among patients with lung carcinoid tumors, no previous study has comprehensively evaluated its impact in different histological subgroups of lung carcinoid tumors. Recently, the International Association for the Study of Lung has showed the clear evidence about impacts of tumor size on outcomes of node-negative disease in the [35]. However, there were insufficient data for TC versus AC to make any conclusions regarding stage in relation to survival. In the current study, tumor size was independently associated with CSS in patients with lung carcinoid tumors. Worse survival rates have been observed among TC patients with tumor size > 20 mm compared to their contemporaries. Similarly, AC patients with tumor size > 40 mm also had worse survival rate than those with tumor size ≥40 mm. These results together convinced the possibility of integrated tumor size into next iteration of the TNM classification/AJCC staging system for better tumor classification. To the best of our knowledge, this is the first population-based study evaluating the impact of tumor size on survival of patients with lung carcinoid tumors according to histological subtypes.

Several limitations in the current study should be noted. First, clinicopathological characteristics derived from the SEER database was the construction basis of our nomogram model, which might have limited the generalization in predicting survival and prognosis of patients with lung carcinoid tumors. Mutational landscape differences between and within histological subtypes of lung carcinoma have challenged the traditional histological classification [36]. Further study will need to incorporate mutational landscape differences into the model for better accuracy in predicting prognosis of lung carcinoid tumors. Unfortunately, our current nomogram model did not take into account the mutational landscape due to unavailability of data. The diagnosis of lung carcinoid tumors requires data on neuroendocrine differentiation, which is recognized by positive immunohistochemical (IHC) stains for Ki-67, mTOR, and chromogranin A [37]. These biomarkers are potential prognostic factors for lung carcinoma. However, as the SEER database did not contain IHC profiles of these markers, the nomogram we built did not incorporate these markers. Also, although we found that radiotherapy is a detrimental factor, we did not further address the prognostic impact of each specific type of radiotherapy, as these relevant data are unavailable in the SEER database. Furthermore, some other important clinicopathological data are not available from the SEER database, including smoking status, family history of cancer, coexisting comorbidities, and performance score. Second, treatment regimens were not available in the SEER dataset, which made our model unable to evaluate the effect of treatment on survival of patients with lung carcinoid tumors. Third, only internal validation was clarified in the current study, although both internal and external validation sets were recommended to validate nomograms. Additional validation study is required in an independent patient population in order to ensure external validation. Finally, we cannot rule out the selection bias due to the retrospective study design. Regardless of these inherent limitations, the SEER database is generally recommended with high quality, and one of the most comprehensive databases suitable to testify our objective.

## Conclusions

We developed a practical nomogram that provided individual predictions of CSS for patients with lung carcinoid tumors using seven clinicopathological factors and two treatment-related factors. Bootstrapping validation of the model confirmed its good performance. This nomogram may help clinicians with prognostic evaluations and with the development of individualized therapy for this aggressive disease. Future prospective studies are required to further determine the impacts of different treatment modalities on the survival of patients with lung carcinoid tumors.

## Data Availability

The data used in this study are available free of charge online at www.seer.cancer.gov on request. All data generated or analyzed during this study are included in this published article.

## Abbreviations

CSS: Cancer-specific survival
C-index: Concordance index
DCAs: Decision curve analyses
ROC: Receiver operating characteristic
TCs: Typical carcinoids
ACs: Atypical carcinoids
TNM: Tumor-node-metastasis
SEER: Surveillance, Epidemiology, and End Results
NSCLC: Non-small cell lung cancer
IHC: Immunohistochemical
